# Supplementary material for: Siboglinidae Tubes as an Additional Niche for Microbial Communities in the Gulf of Cádiz—A Microscopical Appraisal
Source: Microorganisms. 2020 Mar 5;8(3):367. doi: 10.3390/microorganisms8030367 (PMC7143560; doi:10.3390/microorganisms8030367)
Supplement: Supplementary file 1 [file microorganisms-08-00367-s001.zip › supplementary data.docx]

Supplementary data

Worm observed inside its tube in Al Gacel MV sample


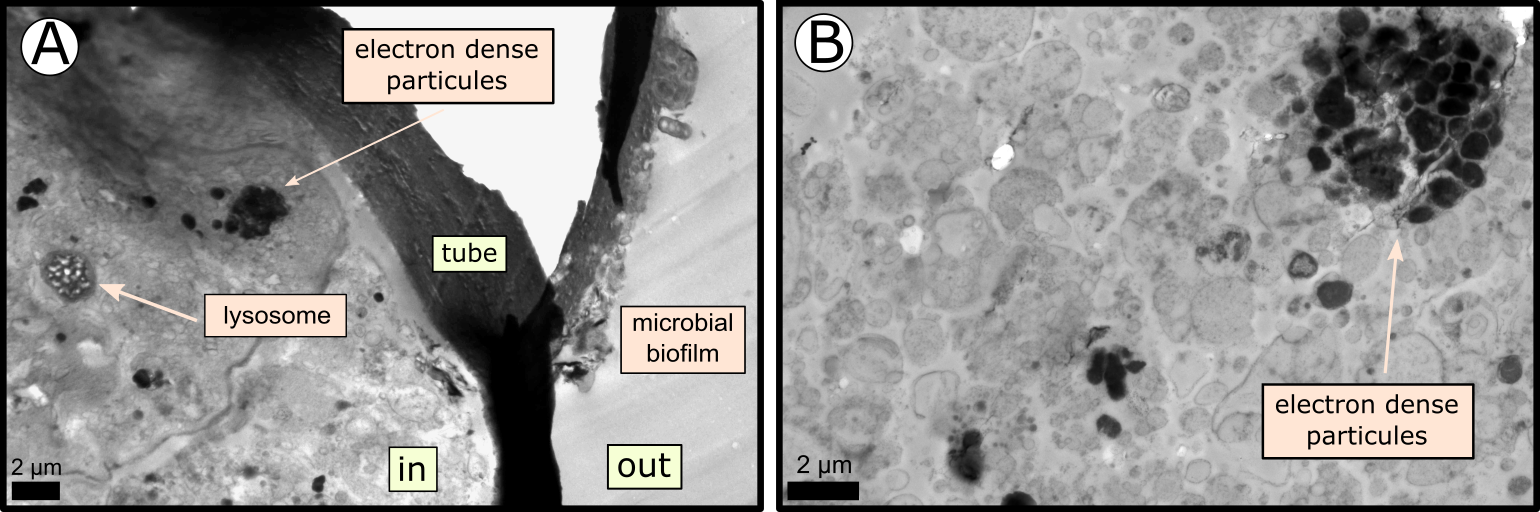


**Figure S1.** **TEM micrographs of siboglinids from Al Gacel MV.**

EDX analysis

El Cid MV specimens expressed high values of iron and calcium along the tube, except on the inside layer (**Figure S2**). Outside layer presented phosphorous and silica (spektrum 4). There were differences in the different internal layer, with variations in phosphorous, aluminum and silica. Magnesium was detected in one of the layers. Filamentous matrix (spektrum 6) was richer in phosphorous and presented lower silica. Layer with silica-balls had pick-signals in silica and aluminum (spektrum 2). The inside layer had no calcium, but maybe it was just not detected. Futhermore, Anastasya MV tubes presented iron and sulfur values homogenous along the tube. The outsider layer revealed also notable presence of silica and alluminium, and picks of calcium locally. Likewise, in Al Gacel MV tube sulfur and silica were homogenous. Outside layers presented locally high picks of sulfur and iron, while internal layer had some aluminum. One internal layer also presented a pick-signal of sulfur. The most inside layer (facing worm), keeps the homogeneity.


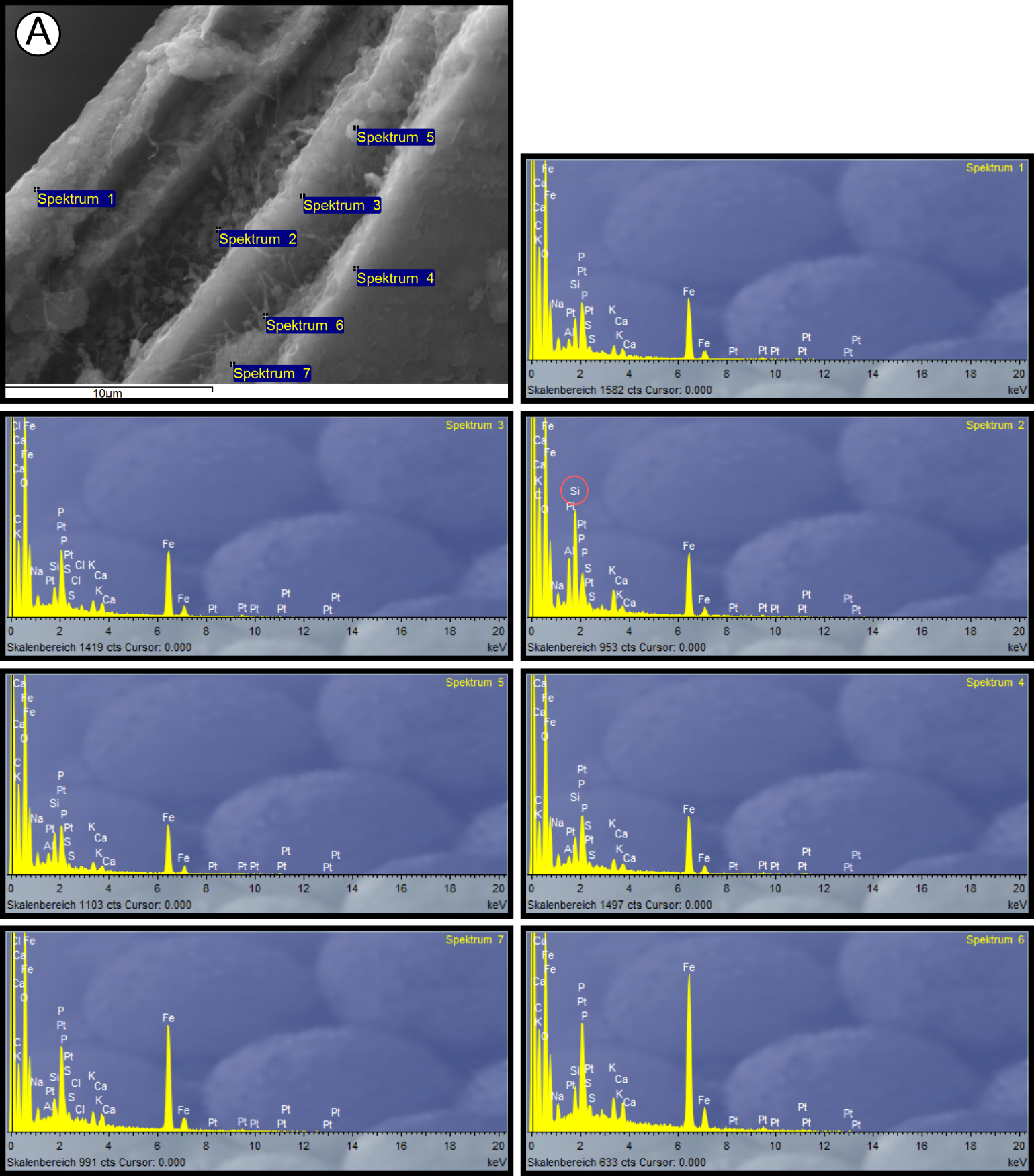


**Figure S2**: EDX analysis performed on the disrupted tube from a specimen from El Cid MV. Notice the pick-signal of silica in Spektrum 2.

Epibionts of Anastasya MV worm


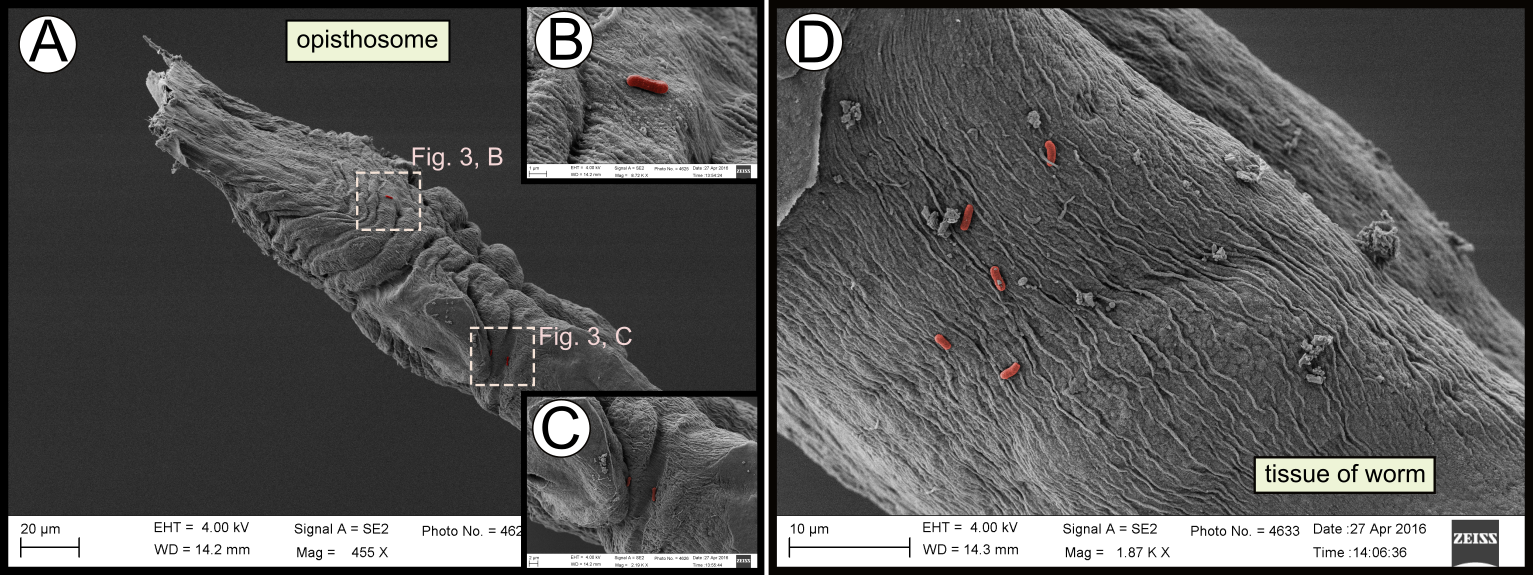


**Figure S3**: SEM micrographs revealed the presence of epibionts on the surface of the worms’ tissue.
