# Supplementary figures and images for: Siboglinidae Tubes as an Additional Niche for Microbial Communities in the Gulf of Cádiz—A Microscopical Appraisal
Source: Microorganisms. 2020 Mar 5;8(3):367. doi: 10.3390/microorganisms8030367 (PMC7143560; doi:10.3390/microorganisms8030367)

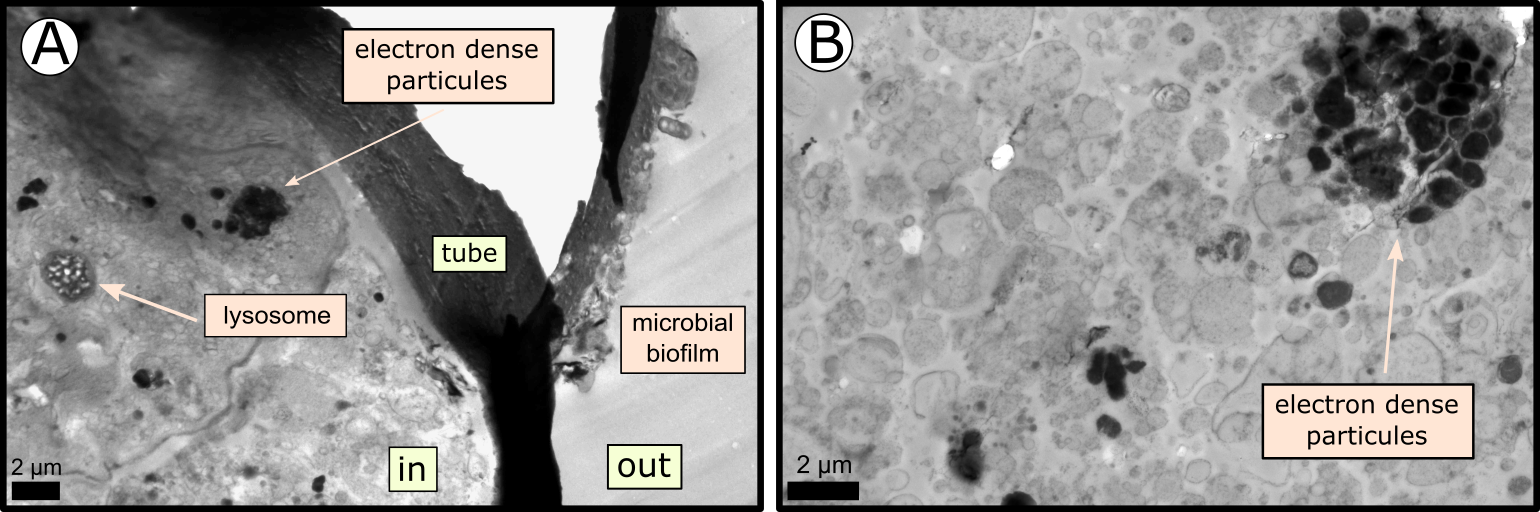

Supplement: Supplementary file 1 [file microorganisms-08-00367-s001.zip › Supplemental Figure S1.png]

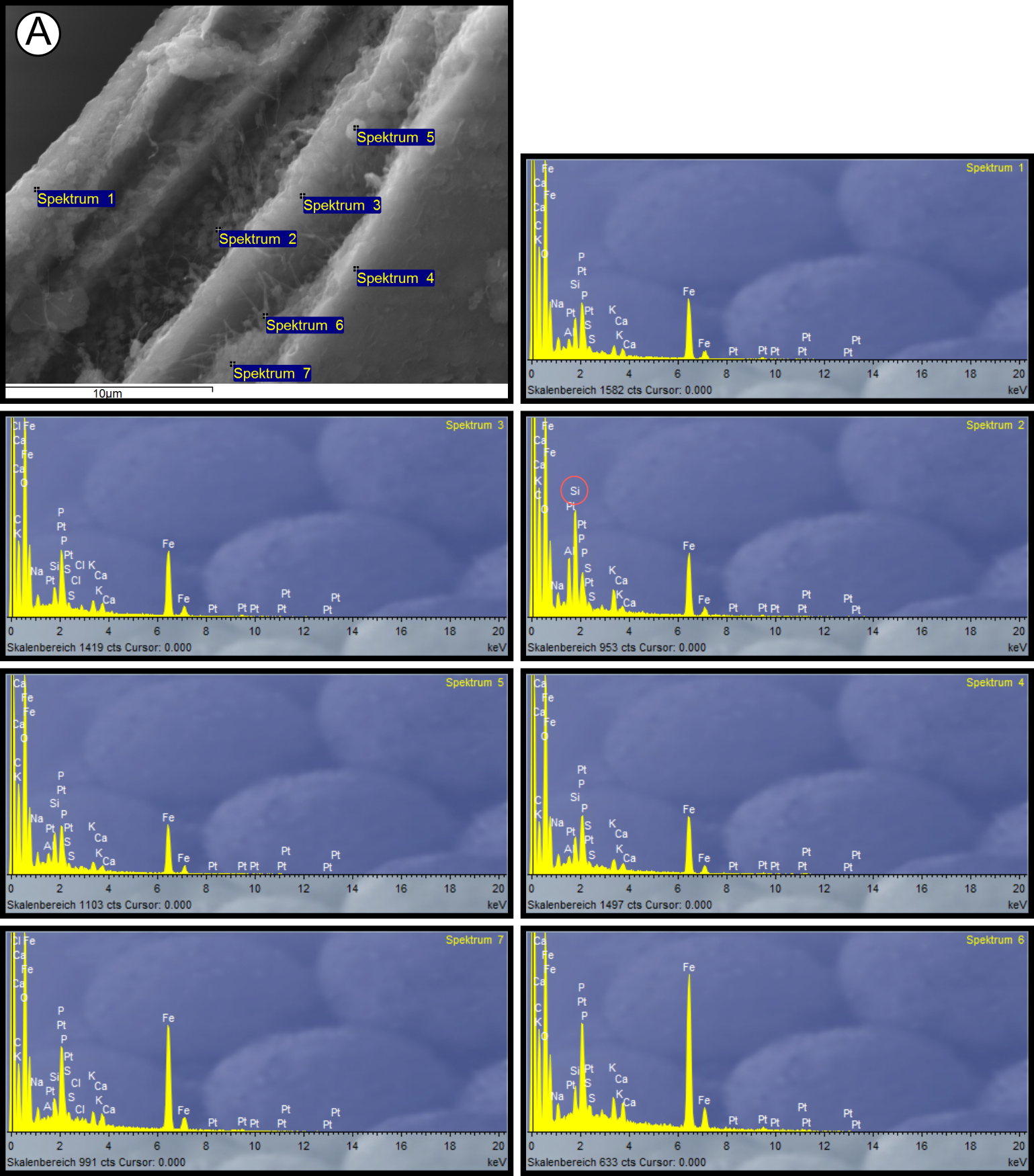

Supplement: Supplementary file 1 [file microorganisms-08-00367-s001.zip › Supplemental Figure S2.png]

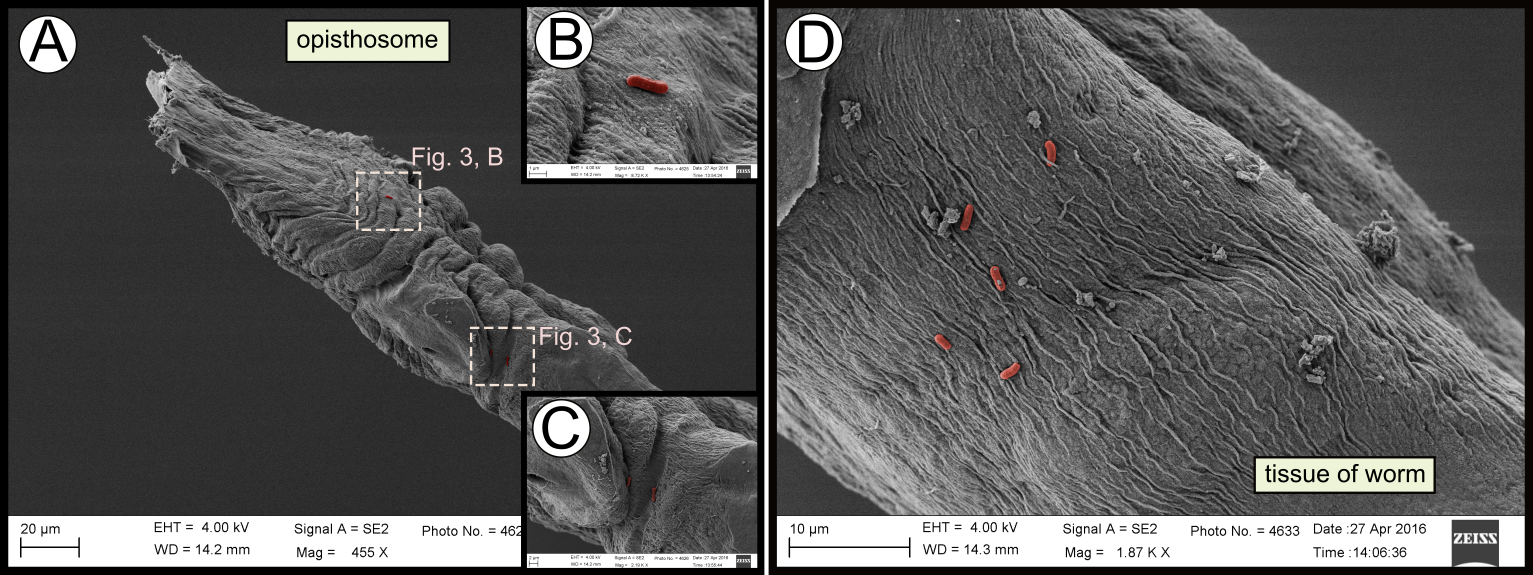

Supplement: Supplementary file 1 [file microorganisms-08-00367-s001.zip › Supplemental Figure S3.png]
